# Holocene winter climate variability in Central and Eastern Europe

**DOI:** 10.1038/s41598-017-01397-w

**Published:** 2017-04-26

**Authors:** Aurel Perșoiu, Bogdan P. Onac, Jonathan G. Wynn, Maarten Blaauw, Monica Ionita, Margareta Hansson

**Affiliations:** 1 Emil Racoviță Institute of Speleology, Romanian Academy, Clinicilor 5, Cluj-Napoca, 400006 Romania; 2 Institute for Advanced Studies, Ștefan cel Mare University, Universității 13, Suceava, 720229 Romania; 3 School of Geosciences, University of South Florida, 4202 E. Fowler Ave., NES 107, Tampa, FL 33620 USA; 4 School of Natural and Built Environment, Queen’s University Belfast, University Road, Belfast, BT7 1NN Northern Ireland UK; 5 Paleoclimate Dynamics Group, Alfred-Wegener-Institute for Polar and Marine Research, Bussestrasse 24, Bremerhaven, D-27570 Germany; 6 MARUM – Center for Marine Environmental Sciences, University of Bremen, Bremen, Germany; 7 Department of Physical Geography, Stockholm University, Svante Arrhenius väg 8, Stockholm, S-106 91 Sweden

## Abstract

Among abundant reconstructions of Holocene climate in Europe, only a handful has addressed winter conditions, and most of these are restricted in length and/or resolution. Here we present a record of late autumn through early winter air temperature and moisture source changes in East-Central Europe for the Holocene, based on stable isotopic analysis of an ice core recovered from a cave in the Romanian Carpathian Mountains. During the past 10,000 years, reconstructed temperature changes followed insolation, with a minimum in the early Holocene, followed by gradual and continuous increase towards the mid-to-late-Holocene peak (between 4-2 kcal BP), and finally by a decrease after 0.8 kcal BP towards a minimum during the Little Ice Age (AD 1300–1850). Reconstructed early Holocene atmospheric circulation patterns were similar to those characteristics of the negative phase of the North Atlantic Oscillation (NAO), while in the late Holocene they resembled those prevailing in the positive NAO phase. The transition between the two regimes occurred abruptly at around 4.7 kcal BP. Remarkably, the widespread cooling at 8.2 kcal BP is not seen very well as a temperature change, but as a shift in moisture source, suggesting weaker westerlies and increased Mediterranean cyclones penetrating northward at this time.

## Introduction

Weather and climate patterns in East-Central Europe (ECE) are strongly influenced by the dynamics of the storm tracks carrying moisture from the North Atlantic Ocean and Mediterranean Sea towards the continent. The strength and position of these storm tracks control short and long-term changes of precipitation amount and distribution^1^, as well as temperature, summer heat, and winter cold waves^2^. Climate models suggest a poleward shift in the position of mid-latitude storm tracks due to increased warming^3^. Under this scenario, central and south Eastern Europe are influenced by subtropical high pressure cells, which heighten risk of droughts^4^ and stronger cyclones^5^. However, these projections are highly uncertain^6^ as models have difficulties capturing present-day dynamics of storm tracks. Therefore, model validation against long-term observational data is needed. Over the past decade, important progresses have been made in reconstructing past hydroclimatic variability in ECE^7, 8^. However, most of these reconstructions are biased towards the warm season; few capture annual climatic information^7, 9, 10^, and none winter climatic conditions, the latter of which is the period most sensitive to changes in moisture sources and precipitation amounts.

**Figure 1:**
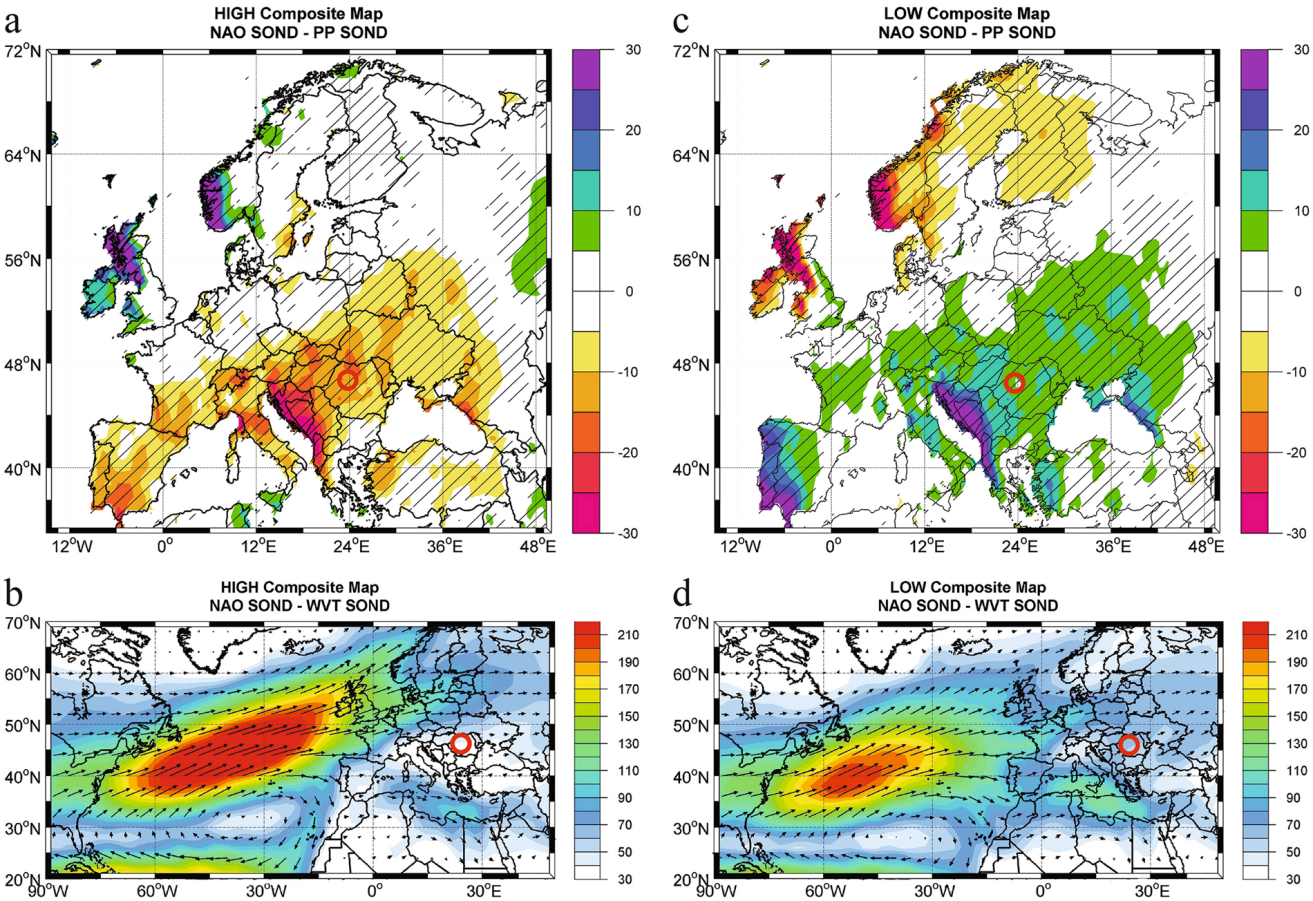
Present day conditions for the positive and negative phase of the winter NAO index. (**a**) Composite map of September-October-November-December (SOND) precipitation (PP) and (**b**) vertically-integrated water vapor transport (WVT) for the years when the SOND NAO index was higher than 1 standard deviation. (**c**,**d**) Same as in (**a**) and (**b**) but for the years when the SOND NAO index was lower than one standard deviation. Units: PP (mm) and WVT (kg m s^−1^). Position of Scărișoara Ice Cave (this study) is marked by a red circle. The figure was produced using Matlab 2014b (http://de.mathworks.com/products/new_products/release2014b.html).

Here we present the first continuous record of changes in temperature and precipitation sources from autumn through early winter (September-December, SOND) in ECE for the past ~10,000 years. It is derived from a precisely ^14^C-dated isotopic record from a cave ice deposit in the Apuseni Mountains (Scărișoara Ice Cave, 46°29′23.64″N, 22°48′37.68″E; Fig. 1a–c). The oxygen and hydrogen isotopic composition (δ^18^O and δ^2^H values) of cave ice reflects changes in air temperature during ice formation (September through December, while deuterium excess (d-excess = δ^2^H-8*δ^18^O) offers ECE’s first terrestrial record of changes in the sources of precipitation and associated shifts in the position of mid-latitude storm tracks.

## Present-day climatic conditions and ice genesis

ECE receives moisture from both the North Atlantic and the Mediterranean Sea, making it a key region to study swings from one source to another. These are determined by atmospheric and oceanic interactions (internally- and externally-forced), on seasonal to millennial time scales. Climate in ECE is strongly seasonal, with hot and dry summers associated with the northward expansion of mid-latitude anticyclonic cells, coupled with cold and wet winters, resulting from complex interplay of southward outbursts of Siberian cold air and northward intrusion of moisture carried by Mediterranean cyclones. The latter originate in the Western and Central Mediterranean, and their path is heavily influenced by the strength of the North Atlantic Oscillation (NAO), one of the principal modes of climate variability in the Northern Hemisphere^11^, defined as the difference of atmospheric pressure between the Icelandic Low and Azores High. The influence of NAO is strong over Europe, especially during the cold season (November through April). When the NAO is in a positive phase (NAO^+^, *i.e*., with a deeper than usual Icelandic Low and/or stronger than usual Azores High), the westerlies carrying moisture are deflected northward, resulting in drier conditions in ECE (Fig. 1a,b). When the NAO is in a negative phase (NAO^−^), the westerlies carry more moisture from the North Atlantic towards South Eastern Europe (Fig. 1c,d).

The mean annual temperature at Scărișoara Ice Cave (SIC) is ~6 °C and precipitation amounts reach 1200 mm/year, with two maxima, one represented by Atlantic-sourced rainfalls in May-June and another of Mediterranean origin in late autumn^12^. The relative contribution of these sources is controlled by the strength of the NAO and East Atlantic-Western Russia (EA-WR) pattern modes of atmospheric variability and the blocking activity of the Siberian High^13, 14^. δ^18^O and δ^2^H values of local precipitation plot along a Local Meteoric Water Line being defined by the equation δ^2^H = 7.9*δ^18^O + 8.14^15^, similar to the Global Meteoric Water Line^16, 17^. The mean d-excess value is close to 10, although higher (between 12 and 15) in autumn, when moisture from the evaporatively ^18^O- and ^2^H-enriched Mediterranean surface waters reaches into ECE^18^.

**Figure 2:**
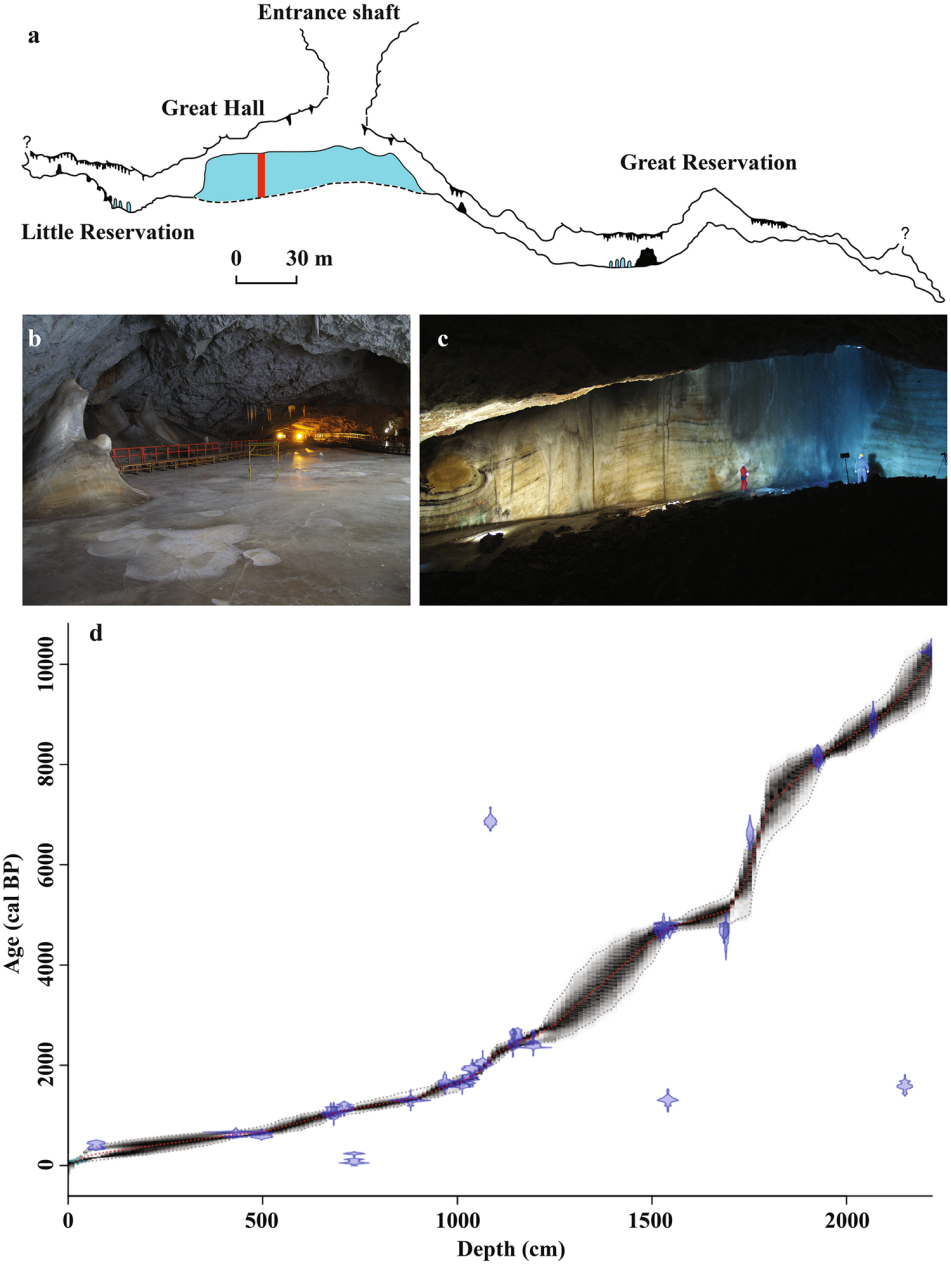
Scărișoara Ice Cave. (**a**) Cross-section of the cave. Red bar marks the position of the drilling site. (**b**) Surface of the ice block. (**c**) Side view of the ice block with visible layering. (**d**) Age-depth model of the ice block. The map in (**a**) is from the archives of the Emil Racoviță Institute of Speleology. The digital version was produced using Adobe Illustrator CS6 (http://www.adobe.com/products/illustrator.html). Photos in (**b**) and (**c**) by Aurel Perșoiu.

Ice in Scărișoara Cave (Fig. 2) forms as seepage water accumulates in a shallow lake on top of the ice block between September and December, and subsequently freezes in winter, to form a layer of ice *ca*. 1–15 cm thick. The cave opens to the surface through two shafts, 47 m and 6 m wide, respectively. The ice block extends from the bottom of the large shaft, continuing under the narrower one. This particular setting allows for direct deposition in the lake on top of the ice block and subsequent incorporation at the base of the newly formed ice layers of surface-derived organic and inorganic matter. Occasional infiltration of seepage water in winter leads to further ice development on top of the already existing one. Spring and summer melting usually removes this ice, so that the ice block mainly consists of ice formed by the freezing of late-autumn and early-winter precipitation. Periods of enhanced melting might have acted in the past, so that the annual layering of the ice is not always preserved^19, 20^. Observation over the past 70 years have regularly shown that the melt water and the detritus it might contain are flowing out of the surface and over the sides of the ice block, so that the newly formed lake in autumn contains no traces of the previous year’s sediments. This has important implications for the age of the ice, as older organic matter released by the melting of ice is not incorporated in the new ice, and thus the ice and the organic matter it contains are of the same age. Based on observations spanning the past 70 years, the long-term dynamics of the ice block is controlled by the strength of winter cooling, with summer melting playing an secondary role. By analyzing the isotopic composition of precipitation, drip and lake water, and resulting ice, our previous studies^21^ have shown that the isotopic signature of the ice block reflects precipitation from autumn through early winter and related climatic information.

## Results and Discussions

The ice accumulation rate increased from 0.13 cm/yr prior to 5 kcal BP (thousands of years before AD 1950) to ~0.4 cm/yr between 5 and 2.5-2 kcal BP, and doubled afterwards to ~0.9 cm/yr (Fig. 2d). The accumulation rate is the result of a complex interplay between water availability and temperature. Thus, irrespective of air temperature variations (as long as they are <0 °C), higher (lower) amounts of precipitation in winter result in more (less) ice formation. In contrast, higher amounts of summer precipitation and increased air temperature will cause enhanced melting of ice. The low accumulation rate in the early Holocene could have thus resulted from a combination of drier/colder winters (with less ice accumulation) and wetter and/or warmer summers (with more ice melting). Contrary, the high accumulation rates after *ca*. 5 kcal BP are likely due to more ice formation in winter (under wetter conditions) and/or less summer ablation (under drier conditions). This hypothesis is supported by proxy (pollen and charcoal) and modeling data^22^, showing early Holocene warm and dry summers, and wet and somewhat colder late Holocene summers. Summer precipitation amounts were relatively constant (low amounts) between 5 and 9 kcal BP, but increased in two steps at 5 kcal BP and after 2.5 kcal BP. Likewise, higher summer temperatures were documented between 8 and 2.4 kcal BP, followed by a cooling tendency after *ca*. 2.4 kcal BP^8, 10^.

**Figure 3:**
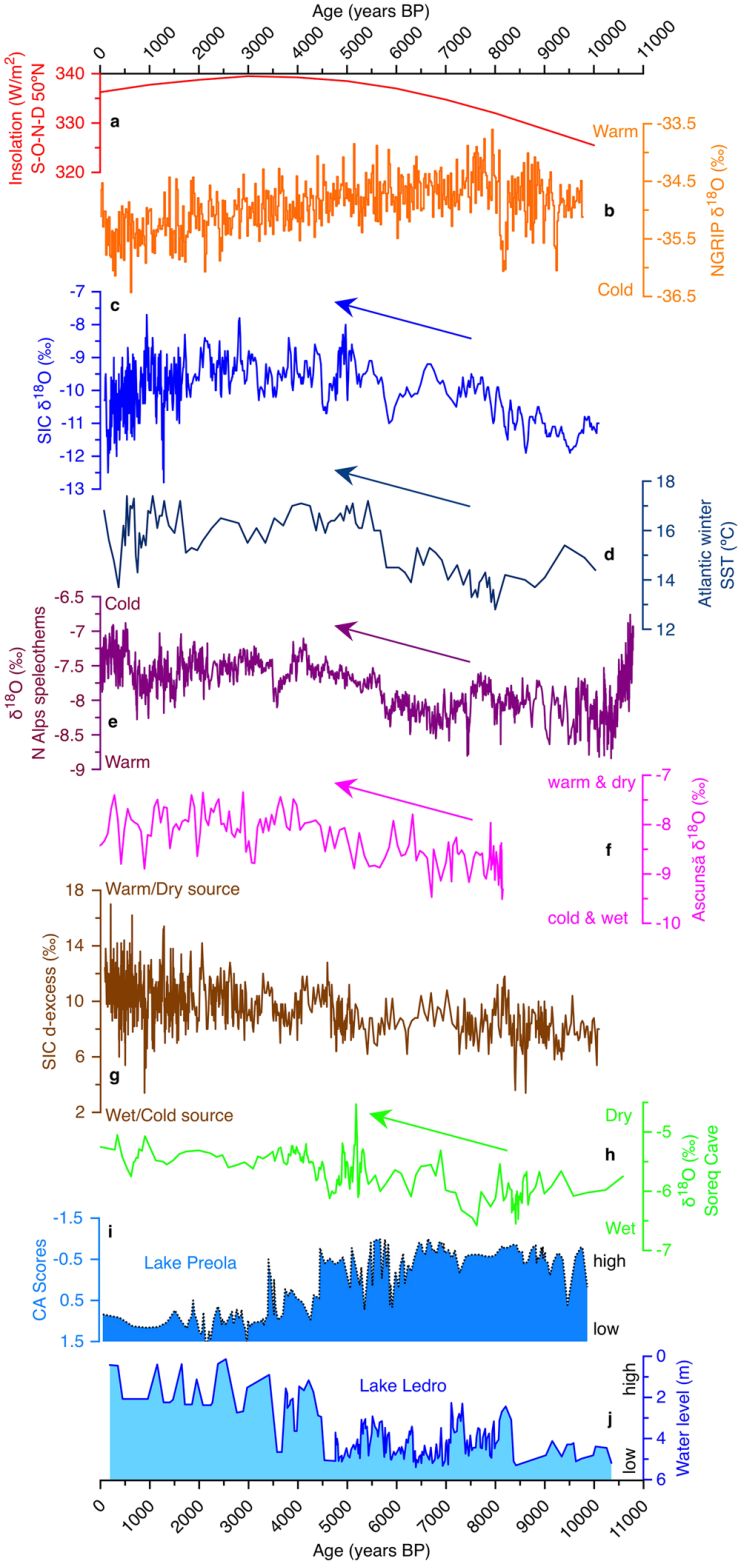
SIC δ^18^O (proxy for autumn through early winter (SOND) temperature) and d-excess (proxy for moisture source) against Northern Hemisphere paleoclimate proxies. (**a**) Insolation during SOND at 50°N^36^. (**b**) NGRIP ice core δ^18^O^37^. (**c**) SIC δ^18^O (this study). (**d**) Atlantic winter sea surface temperature (SST)^38^. (**e**) North Alps speleothem δ^18^O-based winter temperature^39^. (**f**) SW Romania speleothem δ^18^O^7^. (**g**) SIC d-excess record (this study). (**h**) Speleothem δ^18^O in Soreq Cave^31^. (**i**) CA scores (lake level proxy)^26^ at Preola, S Italy, 37°N. (**j**) Water level^26^ at Lake Ledro, N Italy, 45°N.

The stable isotope record from SIC covers almost the entire Holocene, between 10.5 and 0.090 kcal BP (Fig. 3c), closely following the 50°N September-October-November-December (SOND) insolation curve (Fig. 3a). The SIC δ^18^O record shows an early Holocene minimum (−11‰) between 10.4 and 9.25 kcal BP, followed by a gradual and sustained increase to −8‰ until roughly 5 kcal BP, a relatively stable period until 1.5 kcal BP, and a continuous decrease towards the most recent values (−9.5‰) at AD 1860, before the onset of current warming. Combining the winter temperature with that of the summer^22^, it appears that the early Holocene had a more continental climate, with a marked contrast between cold winters and warm summers, whereas during the mid-to-late Holocene, the gradual summer cooling and warming winter resulted in diminishing influence of continental-like climate conditions. The past ~1000 years, up to 1860 AD, when the SIC record stops, both summer and winter were cold compared to the preceding millennia. The SIC d-excess record (Fig. 3g) shows a different pattern of variability, with relatively low values in the early Holocene (average 8.3‰), followed by a continuous increase (of over 3‰) towards present, initiated rather abruptly at 4.7 kcal BP. Both the δ^18^O and d-excess records are punctuated by episodes of abrupt changes. In the d-excess record, the most notable one is at 8.2 kcal BP (Fig. 3g), coincident with a widespread cooling event in the Northern Hemisphere^23^. However, a similar change in the SIC δ^18^O values is not noticeable, possibly because cooling during the 8.2 ka event occurred mostly in summer^24^. The rapid increase in d-excess values during the 8.2 ka event might indicate either increasing evaporation at the moisture source and/or a shift towards more evaporative (and hence warmer) precipitation sources. We suggest that the cooling in the North Atlantic during the 8.2 ka event led to a weakening of the Icelandic Low and of the westerlies, thus allowing for more Mediterranean-derived precipitation to reach SIC. This source would have been warmer, thus explaining the high d-excess values and reduced cooling, as seen in the SIC record.

**Figure 4:**
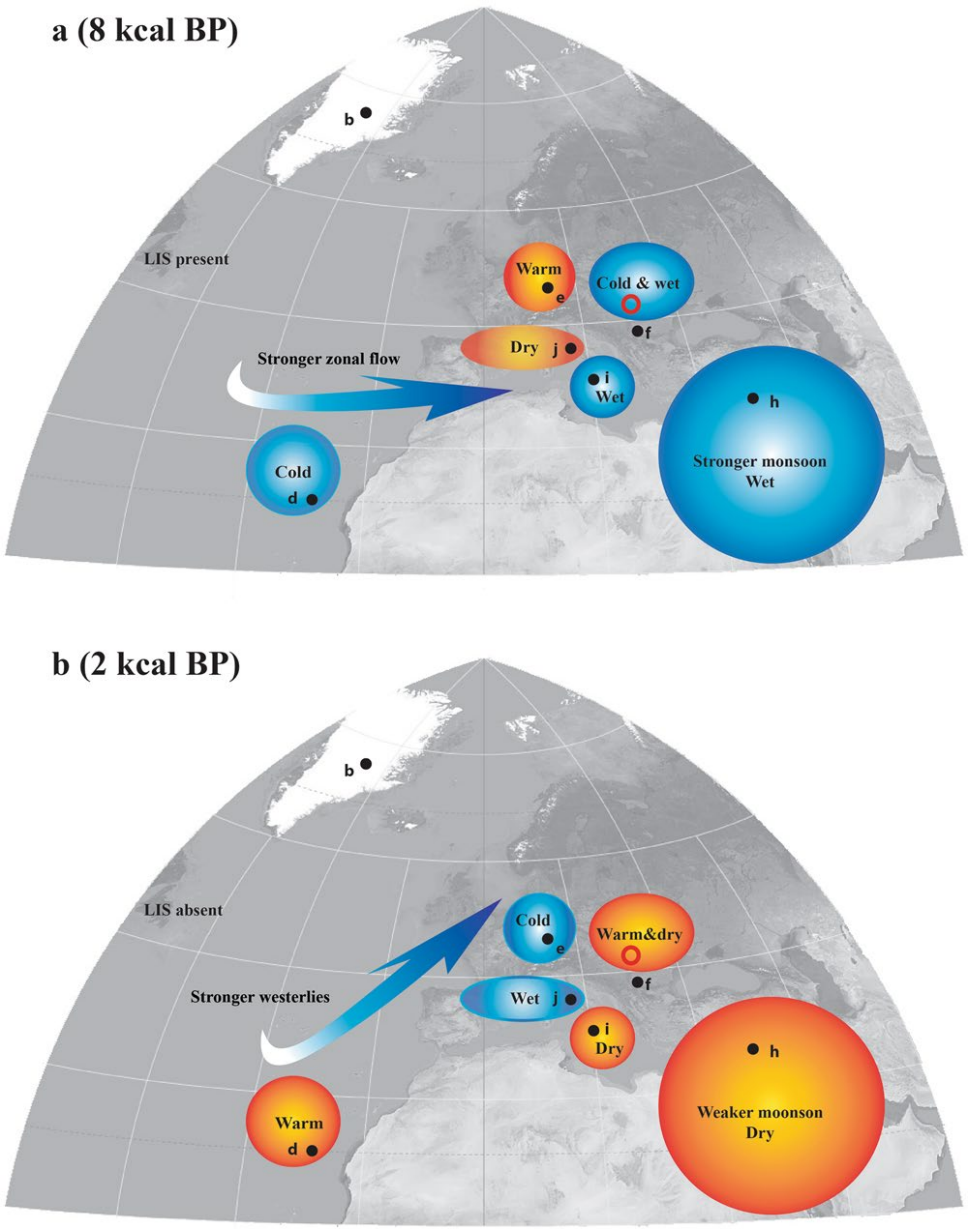
Climatic conditions in Europe during the Holocene. (**a**) At 8 kcal BP (representative for conditions similar to those occurring during the negative phase of the NAO). (**b**) At 2 kcal BP (representative for conditions similar to those occurring during the positive phase of the NAO). Black circles and letters refer to the location of paleoclimatic archives discussed in the text and presented in Fig. 3. Position of Scărișoara Ice Cave (this study) is marked by a red circle. Map produced using Adobe Illustrator CS6 (http://www.adobe.com/products/illustrator.html). The background map is cropped from the world map (Mollweide projection; the imagery is a derivative of NASA’s Blue Marble summer month composite) created by Daniel R. Strebe, using the Geocart software (https://www.mapthematics.com) and available under a CC BY-SA 3.0 license (https://creativecommons.org/licenses/by-sa/3.0/legalcode) at https://commons.wikimedia.org/wiki/File:Mollweide_projection_SW.jpg.

Two prominent features of the SIC record stand out. First, there is a continuous increase (of about 3.2‰) in δ^18^O values between the early Holocene and ~5 kcal BP, followed by relatively steady conditions until ~0.8 kcal BP and a rapid decrease towards AD 1860 (when the record ends). Second, there is a shift towards higher d-excess values, after *ca*. 4.7 kcal BP (Fig. 3c,g). The cold conditions prior to 8 kcal BP conceivably reflect reduced winter insolation and the presence of Laurentide Ice Sheet (LIS) in North America that also affected the atmospheric circulation in the wider North Atlantic realm. The cold North Atlantic sea surface likely induced a weaker than usual Azores High and Icelandic Low, thus leading to weaker and southward-displaced storm tracks. These brought cold and wet weather to southern and central Europe (including the site of this study) and left its northwestern part deprived of moisture and warmth^25–27^. Such conditions are registered by the SIC record, with low δ^18^O and d-excess values both indicating a cold and humid source of precipitation, respectively. Apart from the influence of the North Atlantic cooling, the cold conditions in ECE during the early Holocene could have also resulted from additional advection of polar air, as evidenced by the strengthening of the Siberian High^28^ and increased frequency of outbreaks of cold air from the northeast over the Aegean Sea^29^. Taken together, these data suggest that during the early to mid-Holocene (prior to *ca*. 4.7 kcal BP) weather patterns in the wider North Atlantic basin were similar to those occurring during NAO^−^ phases, with dry northwestern and western Europe and wet southeastern Europe (Fig. 4a). Additional support to this hypothesis comes from northeastern North America, where varve thickness data imply weaker westerlies (as expected during NAO^−^ conditions) in the early Holocene, with an increasing trend towards 4–5.5 kcal BP^30^. Strengthening of westerlies and strong zonal flow from 10 to 4.7 kcal BP indicates a shift from NAO^−^ to NAO^+^ in the North Atlantic, the latter becoming dominant since ~4.7 kcal BP. The SIC δ^18^O record (Fig. 3c) shows that after peaking at ~5 kcal BP, winter temperatures remained high, but variable until *ca*. 0.6 kcal BP, when they started to decrease, in phase with the onset of the Little Ice Age cooling. The increase in d-excess (Fig. 3g) suggests either stronger evaporative conditions at the moisture source, or a shift towards a highly evaporative moisture source, such as the Mediterranean Sea. Paleoclimatological data for winter conditions in Europe during the 1–5 kcal BP interval show wetter conditions in western and northern Europe and in the Atlantic (Fig. 4b), and drier conditions in southern and eastern Europe, all suggesting a positive NAO^-^ like state. Furthermore, lake levels in Europe (Fig. 3i,j) show contrasting trends, higher in northwestern Europe and lower in the East-Central Mediterranean Sea, opposite to conditions in the early to mid-Holocene, when they were higher in the East-Central Mediterranean Sea and lower in northwestern Europe^26^. In the Eastern Mediterranean climatic conditions inferred from speleothem δ^18^O (here a proxy for winter climatic conditions) suggest a wet early Holocene and rather dry conditions after ca. 4.7 kcal BP^31^ (Fig. 3h). Taken together, these climatic conditions resemble those encountered during NAO^+^ conditions (Fig. 4b), indicating that around 5 kcal BP, a major change in the NAO phase occurred, shifting it towards a semi-permanent state similar to the NAO^+^ lasting until 0.7–1 kcal BP^32^. An important feature of the imprint of NAO variability on Eastern European climate is that the northward deflection of the Atlantic storm tracks during NAO^+^ conditions allow for a consequent northward expansion of the Mediterranean-type climate, mostly in the form of more frequent cyclones traveling across the Balkan Peninsula towards NE Europe. The warm conditions in SE Europe and over the Eastern Mediterranean could have led to more evaporative conditions above the sea surface and subsequent higher d-excess values that, combined with the increased number of cyclones crossing towards the NE could explain both higher winter temperatures (as indicated by the δ^18^O SIC record) and more evaporative precipitation source. Further support for this hypothesis comes from the accumulation rate in Scărișoara Ice Cave, which shows an increase at ~5 kcal BP and ~2 kcal BP, coincident with increases in d-excess values in the ice core. In western Romania, Mediterranean cyclones are active mostly in late autumn and early winter^33^; thus intensification in their activity would explain the increase in d-excess (delivery of more precipitation from the evaporative Mediterranean Sea) and accumulation rate (more precipitation reaching the cave in the ice forming season). The increase in d-excess values that occurred after ca. 4.7 kcal BP, was interrupted at ~4.2 kcal BP by a shift towards lower values that lasted until ~2.5 kcal BP (interrupted by brief episodes of higher values). In the context of the above scenario, the lower (although highly variable) d-excess values during this interval imply a return towards stronger zonal circulation, suggesting negative NAO− like conditions, similar to the conditions documented in SW Greenland^32^.

## Conclusions

This is the first study to provide a long term, high resolution record of winter temperature and moisture sources changes in Europe, derived from the stable isotopic composition of underground glaciers.

Throughout the Holocene, the subterranean ice block in Scărișoara Ice Cave responded sensitively to changes in both winter temperature and moisture source. During this time period, winter temperature in ECE was mainly controlled by insolation changes. The interplay between insolation variability, SST changes in the North Atlantic, and the influence of the lingering Laurentide Ice Sheet modulated the dynamics of large-scale atmospheric circulation. Conditions mimicking those occurring during NAO^−^ prevailed in the course of the early Holocene, followed after ~4.7 kcal BP by a shift towards a predominance of NAO^+^ type atmospheric circulation, that lasted with interruptions (most notably, between 4.2 and 2.5 kcal BP) until present. This shift led to more Mediterranean cyclones penetrating further NE into the European mainland, leading to the differentiation between the Atlantic-dominated European climate in W and NW Europe, and a Mediterranean climate in S, SE, and East-Central Europe.

## Methods

### Drilling and sampling

In February 2003, a 22.53 m long core was drilled to the bedrock, using a 10-cm diameter drill, driven by a 220 V engine^34^. A total of 56 ice cores, ranging in length between 21 and 63 cm, were recovered and transported in frozen state to Stockholm University, Sweden. In the cold laboratory, the ice core was cut lengthwise, half of it being archived. The remaining half was cleaned by removing the outer layer of ice (0.5–1 cm thick), and subsequently cut in 1–2 cm wide samples that were allowed to melt at room temperature and stored in 20 ml high-density polyethylene flasks before analyses. Organic matter (leaf pieces and pine needles) was handpicked from the ice core for ^14^C dating.

### Dating and depth-age model construction

A total of 35 samples were submitted for ^14^C dating to the Poznan Radiocarbon Laboratory (Poland), of which six had not enough carbon for precise age determination. Of the resulting^29^ determined ages, three were obviously contaminated (two with young organic matter, and one with old carbon, possibly by incorporating “infinite” aged carbon from the cave’s host-rock). The SIC core chronology (Fig. 2d) was constructed using the Bayesian software Bacon^35^, which assumes that accumulation rate is always positive and can vary gradually from depth to depth. Historical observations have shown that between AD 1863 and 1982, enhanced melting and related changes in the geometry of the ice block led to the loss of ~100 cm of ice. Based on annual ice accumulation rates derived independently^19^ of the current one (between 0.9 and 1.6 cm/year, with a mean value of ~1.3 cm/yr), we estimated an age of AD 1860 ± 20 (similar to 90 cal BP) for the top of the ice core. The raw ^14^C data are included in Supplementary Dataset 1, and the results of modeling in Supplementary Dataset 2.

### Stable isotope analyses

Stable isotope analyses were performed at the School of Geosciences, University of South Florida, using the equilibration method on a Thermo Delta V Advantage Isotope Ratio Mass Spectrometer. The results were normalized to the VSMOW-SLAP scale and reported in the conventional δ notation, in ‰ (per mil) against VSMOW (Vienna Standard Mean Ocean Water), with precision better than ±0.2‰ and ±1‰ for δ^18^O and δ^2^H, respectively (Supplementary Dataset 3).

## Electronic supplementary material

Dataset 1

Dataset 2

Dataset 3
